# The Use of Glucocorticoids in Lupus Nephritis: New Pathways for an Old Drug

**DOI:** 10.3389/fmed.2021.622225

**Published:** 2021-02-16

**Authors:** Juan M. Mejía-Vilet, Isabelle Ayoub

**Affiliations:** ^1^Department of Nephrology and Mineral Metabolism, Instituto Nacional de Ciencias Médicas y Nutrición Salvador Zubirán, Mexico, Mexico; ^2^Division of Nephrology, Department of Internal Medicine, The Ohio State University Wexner Medical Center, Columbus, OH, United States

**Keywords:** glucocorticoids, lupus nephritis, systemic lupus erythematosus, prednisone, methylpredisolone, steroids, adverse effect

## Abstract

Glucocorticoids therapy has greatly improved the outcome of lupus nephritis patients. Since their discovery, their adverse effects have counterbalanced their beneficial anti-inflammatory effects. Glucocorticoids exert their effects through both genomic and non-genomic pathways. Differential activation of these pathways is clinically relevant in terms of benefit and adverse effects. Ongoing aims in lupus nephritis treatment development focus on a better use of glucocorticoids combined with immunosuppressant drugs and biologics. Newer regimens aim to decrease the peak glucocorticoid dose, allow a rapid glucocorticoid tapering, and intend to control disease activity with a lower cumulative glucocorticoid exposure. In this review we discuss the mechanisms, adverse effects and recent strategies to limit glucocorticoid exposure without compromising treatment efficacy.

## Introduction

Cortisone (“compound E” or *17-hydroxy-11-dehydrocorticosterone*) was identified in the 1930's by Edward Kendall and Tadeusz Reichstein, and later purified and synthesized in the 1940's. Compound E had strong anti-inflammatory effects but also potent mineralocorticoid effects which manifested as fluid retention, hypertension, and hypokalemia. Compound E was first applied for treatment in 1948 by Philip Hench. At that time, a young woman with severe rheumatoid arthritis would become the first patient treated with cortisone. The anti-inflammatory effects of the cortisone were remarkable but so were the adverse effects (1, 2).

Subsequently, other glucocorticoid (GC) preparations were developed for treating autoimmune diseases, including systemic lupus erythematosus (SLE) (3). The use of these anti-inflammatory steroids in lupus nephritis (LN) dramatically improved survival. For example, survival was 17% at 5 years in the pre-glucocorticoid era, but 55% at 5 years after introduction of glucocorticoids (4, 5). The addition of immunosuppressive drugs to GC, and later on, the development of biologic drugs, have transitioned LN management to one focused on improving kidney outcomes while minimizing adverse events. In this review, we discuss the use of GCs from mechanisms, adverse events to management of lupus nephritis, and current strategies to limit toxicity of these drugs.

## Mechanism of Action: The Clinical Relevance of The Genomic and Non-Genomic Mechanisms

Glucocorticoids are involved in regulatory processes throughout the body, such as energy and lipid metabolism, and adaptation to stress. Two of their most important effects are their strong anti-inflammatory and immunosuppressive effects, evident at concentrations above the physiological glucocorticoid levels (6).

**Figure 1:**
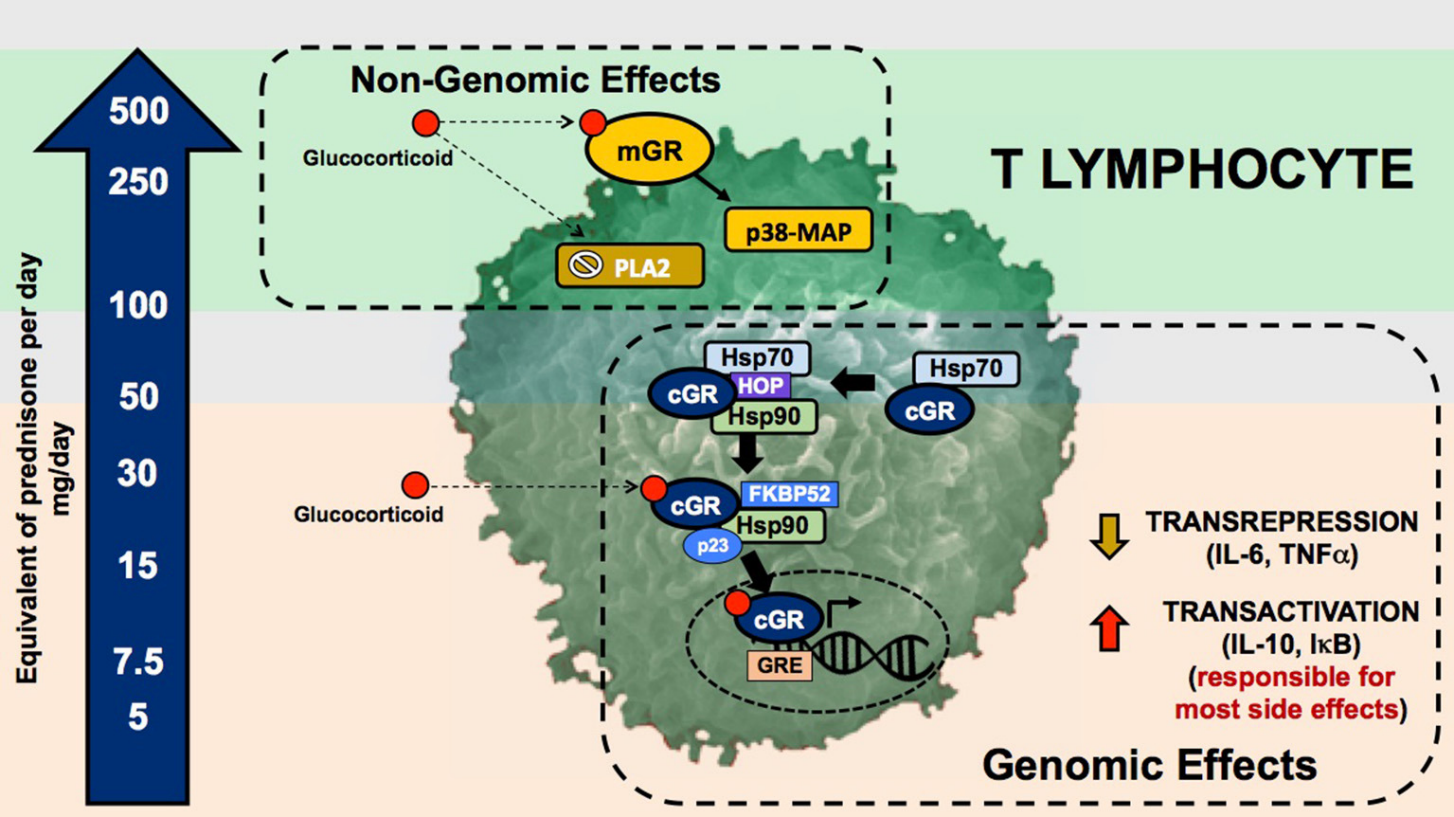
Genomic and non-genomic mechanisms of glucocorticoids. Glucocorticoid genomic pathway is mediated through the cytoplasmic glucocorticoid receptor (cGR) leading to the mechanisms of gene transactivation and transrepression. The non-genomic pathway is mediated through the membrane glucocorticoid receptor (mGR), inhibition of the phospholipase A2, and changes in cell membranes. The arrow in the left depicts the dose of prednisone required to activate these pathways. The upper and lower gray zones represent the doses were genomic (lower gray zone) and non-genomic (upper gray zone) are fully saturated without added benefit and with higher incidence of adverse effects. mGR, membrane glucocorticoid receptor; PLA2, phospholipase A2; cGR, cytoplasmic glucocorticoid receptor; GRE, glucocorticoid response element; Hsp70·HOP·Hsp90, multiprotein complex including chaperones such as heat shock proteins and the glucocorticoid receptor; Hsp90·FKBP52·p23, multiprotein complex including chaperones, co-chaperones, and the glucocorticoid receptor.

Glucocorticoids and synthetic glucocorticoids have two mechanisms of action: the genomic and non-genomic mechanisms (Figure 1) (7). Genomic mechanisms are activated after GC, as lipophilic molecules, cross the cell membranes and bind to the multiprotein complex of chaperones (e.g., Hsp40, Hsp56, Hsp70, and Hsp90), immunophilins that act as co-chaperones (e.g., p23, FKBP51, FKBP52), and the intracellular cytoplasmic glucocorticoid receptor (cGR). After binding and subsequent dissociation from these proteins, the complex GC-cGR translocates to the nucleus and binds to DNA binding sites known as *glucocorticoid response elements*. The final result is a decreased transcription of genes encoding inflammatory cytokines (e.g., interleukin-6, interleukin-8, tumor necrosis factor-a), a process known as *transrepression*; and an increased transcription of anti-inflammatory genes (e.g., interleukin-10, IκB, annexin A1), known as *transactivation* (8).

Genomic mechanisms are generally evident 30 min after GC administration. By contrast, a second type of non-genomic mechanisms produce effects within minutes after the administration. These non-genomic effects are mediated through changes in cellular membranes, inactivation of the phospholipase A2 enzyme, and interaction with membrane glucocorticoid receptors (mGR). Second messengers include kinases, such as the p38 MAP kinase. The final effect is decreased lymphocyte activity and proliferation (9).

**Figure 2:**
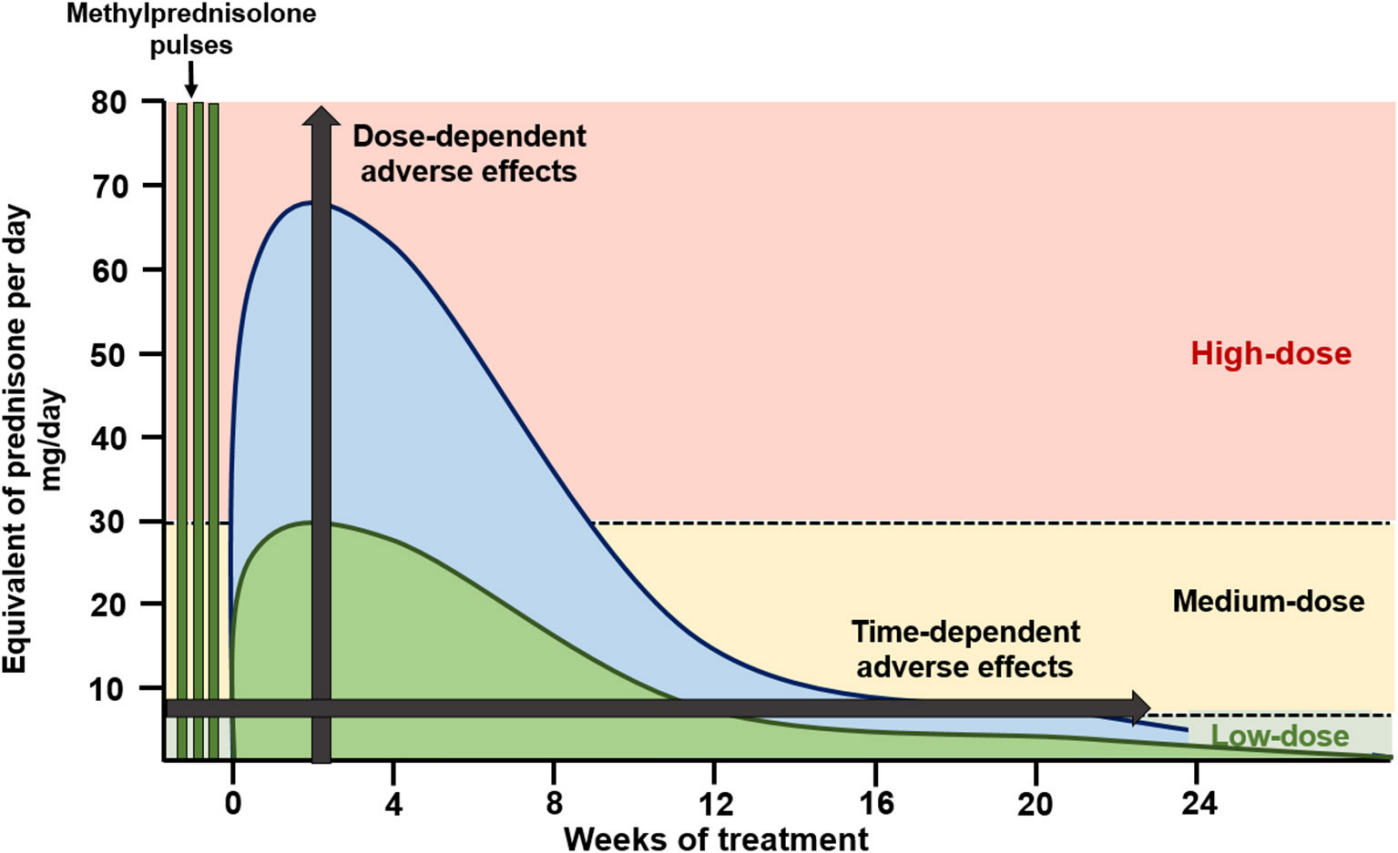
Glucocorticoid dosing in induction of remission schemes. High-dose oral glucocorticoid schemes (blue) apply starting doses of oral glucocorticoids at 0.8–1.0 mg/kg/day, with slow tapering, reaching low glucocorticoid doses by 24 weeks of therapy. Recent schemes (green) apply methylprednisolone pulses followed by medium starting doses of oral glucocorticoids (<0.5 mg/kg/day) with a faster tapering, reaching low glucocorticoid doses by 12 weeks of therapy.

Identification of genomic and non-genomic mechanisms is clinically important due to the differential adverse effect profile and differential activation exerted by currently used glucocorticoid dosages and preparations (Figure 1). Genomic effects are activated with low (<7.5 mg prednisone equivalent per day) to moderate (7.5–30 mg prednisone equivalent per day) GC doses, and cGRs are progressively saturated with high-doses above 30 to 50 mg per day (10). From this pharmacologic concept, prednisone doses above 50 mg per day approach the ceiling of cGR saturation, with limited additional anti-inflammatory benefit, yet increasing the risk for adverse effects. As will be further discussed, some adverse effects, such as avascular bone necrosis, are dependent on the peak GC dose and duration of high-dose exposure (tapering speed) (Figure 2).

Non-genomic mechanisms are activated with very-high GC dosages, such as those reached with methylprednisolone pulses. This activation starts at prednisone dosages of 100 mg, and reaching its maximum around 250 to 500 mg. In contrast to effects mediated by genomic mechanisms, non-genomic mechanisms are thought to be associated with less adverse effects, at least in part due to the short duration of administration (11).

The relative activation of these genomic and non-genomic pathways differs among different GC preparations. For example, dexamethasone and methylprednisolone activate the non-genomic pathway at a 3-fold greater rate than prednisone (12). Different GC preparations also differ in potency (expressed relative to hydrocortisone), mineralocorticoid effects, and duration of suppression of the hypothalamic-pituitary-adrenal axis (13). Other factors, such as time of administration (less suppression when administered in the morning) and their chronopharmacology, contribute to the degree of GC-axis suppression and in consequence to the severity of adverse effects, but are beyond the scope of this review (14).

Understanding these mechanisms is important to develop strategies to limit GC toxicity. As shown in Figure 2, GC administration strategies used in recent clinical trials have included intravenous methylprednisolone pulses, which activate non-genomic pathways, followed by lower peak oral GC dosages and a faster tapering of oral GCs. This strategy aims to maintain treatment efficacy while limiting GC-related adverse effects.

## Glucocorticoid-Related Adverse Events

Both disease activity and glucocorticoid exposure have been associated with organ damage in SLE (15, 16). As patients with higher degree of disease activity are usually treated with higher GC doses, many of the reported studies suffer of confounding by indication (i.e., patients with more severe activity are administered higher GC doses). Also, as damage is frequently measured through indices that group several manifestations [e.g., the SLICC/ACR damage index (SDI)], it is difficult to distinguish organ damage caused by prednisone from that caused by disease activity or concomitant immunosuppressive medications (17). Finally, many studies also suffer from time bias, as the contribution of disease activity to damage is usually higher at earlier stages, while GC-related damage is greater at later stages (17).

Organ damage occurs in 50% of patients with SLE within 5-years of SLE diagnosis (18), with reported increased risk of 2.8% for each 1 mg prednisone per day (19). Organ damage has been reported to be minimized by achieving disease remission (15, 20), and by using maintenance doses of prednisone lower than 6.0 to 7.5 mg per day (21–23).

GC-related adverse effects have also been classified into those related to high dosing over a short period of time, and those related to cumulative GC doses. Table 1 summarizes the reported GC adverse effects according to the use of intravenous methylprednisolone pulses, the peak oral-GC dose, the duration of exposure to high-GC doses, and the GC cumulative dose.

**Table 1:** Reported associations between glucocorticoid (GC) administration and adverse effects.

**Methylprednisolone pulses**	**High GC doses**	**Longer time under high GC doses**	**Cumulative GC dose**
Acute cardiovascular events	Cardiovascular events	Cardiovascular events	Cardiovascular events
Acute cerebrovascular event	Cerebrovascular events	Bacterial and opportunistic infection	Hypertension
Uncontrolled glucose	Insulin resistance	Insulin resistance	Insulin resistance
Uncontrolled hypertension	Cushingoid features	Cushingoid features	Skin thinning, bruising
	Peptic ulcer disease	Weight gain	Hypertension
	Myopathy	Dyslipidemia	Osteoporosis and vertebral fractures
	Mood disorders	Glaucoma	Sleep disorders
	Psychiatric	Osteoporosis	Avascular necrosis
	Sleep disorders		
	Avascular necrosis		

## Infections

Infections have been frequently associated with the peak dose of GC and the duration of exposure to high GC doses. Infections continue to be a major cause of hospitalization and mortality in SLE (24–26). An increased incidence of these infections occurs in patients with kidney disease (27). Although bacterial infections in lungs, skin, and urinary tract are far more frequent (25, 28, 29), the risk for both bacterial and opportunistic infections increases progressively with the use of medium- to high-dose of GC (30–33). The risk of infections associated with high-dose GC administration seems to be independent of the use of other immunosuppressive medications (21).

Studies of infections with administration of methylprednisolone pulses have also been confounded by indication, due to the traditional administration of this treatment in combination with other aggressive immunosuppressive regimens to sicker patients (32, 34). Some studies suggest that the risk of infection is lower with the use of methylprednisolone pulses of less than 1.5 g in total (35, 36). It has also been hypothesized that the shorter duration of pulse therapy (3–5 days) may limit the prolonged suppression of T-cell responses, which usually peaks after 21 days of GC administration (37). Therefore, methylprednisolone pulses of less than 1.5 g in total followed by reduced oral GC may potentially decrease the incidence of steroid induced infections. Additional preventive measures include vaccination and the use of prophylactic antibiotics and antivirals when indicated (38, 39).

## Bone Disease

Avascular bone necrosis occurs in 5–15% of patients with SLE. It is most commonly found in the femoral head, but may occur in other weight-bearing joints, and may occur bilaterally (40–42). The pathophysiology of avascular bone necrosis is not fully understood and suggested mechanisms are reviewed elsewhere (43). As for infections, avascular bone necrosis has been reported to occur more frequently in association with lupus nephritis (44, 45). Also, it has been associated with GC pulse therapy (46), the peak initial GC dose (47, 48), and the high cumulative GC doses in the first months of treatment (40, 49).

The prevalence of osteoporosis in SLE is 10 to 20%, with up to 20% of patients experiencing vertebral fractures (50). Glucocorticoids increase bone resorption and reduce bone formation. The former is more pronounced in the first months of steroid use while the latter becomes predominant with chronic GC use (51). Osteoporosis and vertebral fractures have been associated with higher GC doses, cumulative doses, and prolonged administration (52). The risk of osteoporotic fractures has been estimated to increase 4.2% for each 1 mg per day of prednisone (19). As bone loss develops over a long-time, many studies with short follow-up fail to assess the impact of GC therapy on bone density. Assessment of risk for fractures and of the need for concomitant preventive therapies including calcium, vitamin D, and bisphosphonates are recommended for all patients on GC therapy and are reviewed elsewhere (53, 54).

### Metabolic Disease

Long-term and high-dose GC therapy are associated with pro-atherogenic disturbances that characterize the metabolic syndrome (55). This syndrome occurs in 30 to 40% of patients with SLE and has been associated with higher disease activity, past or present history of LN, and higher oral doses of GCs. Its prevalence varies according to age and ethnicity as expected (56, 57).

Insulin resistance increases in patients with SLE on oral GC above 7.5 mg per day (58). Furthermore, the risk of diabetes increases 2- to 4-fold in non-diabetic patients with SLE, especially with increasing years of chronic GC use (59–61). In patients with pre-existing diabetes, exacerbation of the disease is particularly severe in patients with poor glycemic control at baseline (62, 63).

Hypertension is common in SLE and LN patients, with a prevalence up to 70% when assessed by 24-h blood pressure monitoring (64). Acute exacerbation of hypertension is frequent during pulse GC therapy. Although hypertension during an active LN is mediated by salt-sensitive mechanisms (65), the risk of hypertension has been also reported to be higher in patients exposed to GC, and has been associated with the duration of exposure and the daily dosage of GCs (61, 66, 67).

Glucocorticoids contribute to weight gain by increasing the appetite for high caloric, high fat food intake (68, 69). The weight gain is characterized by central hypertrophy of adipose tissue with concomitant thinning of peripheral subcutaneous adiposity, providing a lipodystrophic appearance (Cushingoid phenotype) (70). Up to 60–70% of patients prescribed long-term GCs report weight gain (52), and this effect has been associated with doses of GC above 5 mg per day (52, 61).

## Cardiovascular Disease

It is known that the incidence of cardiovascular events is increased in SLE, particularly, in patients with lupus nephritis and chronic kidney disease (23). Although it is difficult to differentiate the effect of disease activity, traditional risk factors, and treatment-related factors; the use of medium- to high-dose GCs has been associated with increased cardiovascular events, subclinical atherosclerosis such as carotid intima-media thickening, severity of coronary calcifications, and severity of arterial stiffness (71, 72). The risk of cardiovascular events is estimated to increase 5-fold in SLE patients taking >20 mg per day of prednisone (23), and 3-fold in those who develop cushingoid features (73). Cardiovascular events may be reduced by administering lower peak GC doses, faster GC tapering, and by limiting cumulative dose. In fact, reductions in cumulative oral GC were associated with lower incidence of cardiovascular events in a reported cohort study (74).

## Strategies To Minimize Glucocorticoid Exposure During The Induction Phase of Treatment

The treatment of lupus nephritis has been traditionally divided into an induction phase of intense immunosuppression, aimed to quickly suppress inflammation, followed by a prolonged maintenance phase, directed to consolidate response and to prevent disease flares (75). For the induction phase, current guidelines recommend the use of medium to high-dose GCs, combined with an immunosuppressant such as mycophenolate mofetil, cyclophosphamide, and more recently, calcineurin inhibitors (38, 39). Next, we describe strategies aimed to reduce exposure while keeping treatment efficacy. These strategies have 3 main objectives: (1) reducing the peak GC dose, (2) reducing the duration of exposure to high-dose GC via a faster GC tapering, and (3) limiting the cumulative dose from prolonged administration.

## The Use of Intravenous Methylprednisolone Pulses To Limit Glucocorticoid Exposure

**Figure 3:**
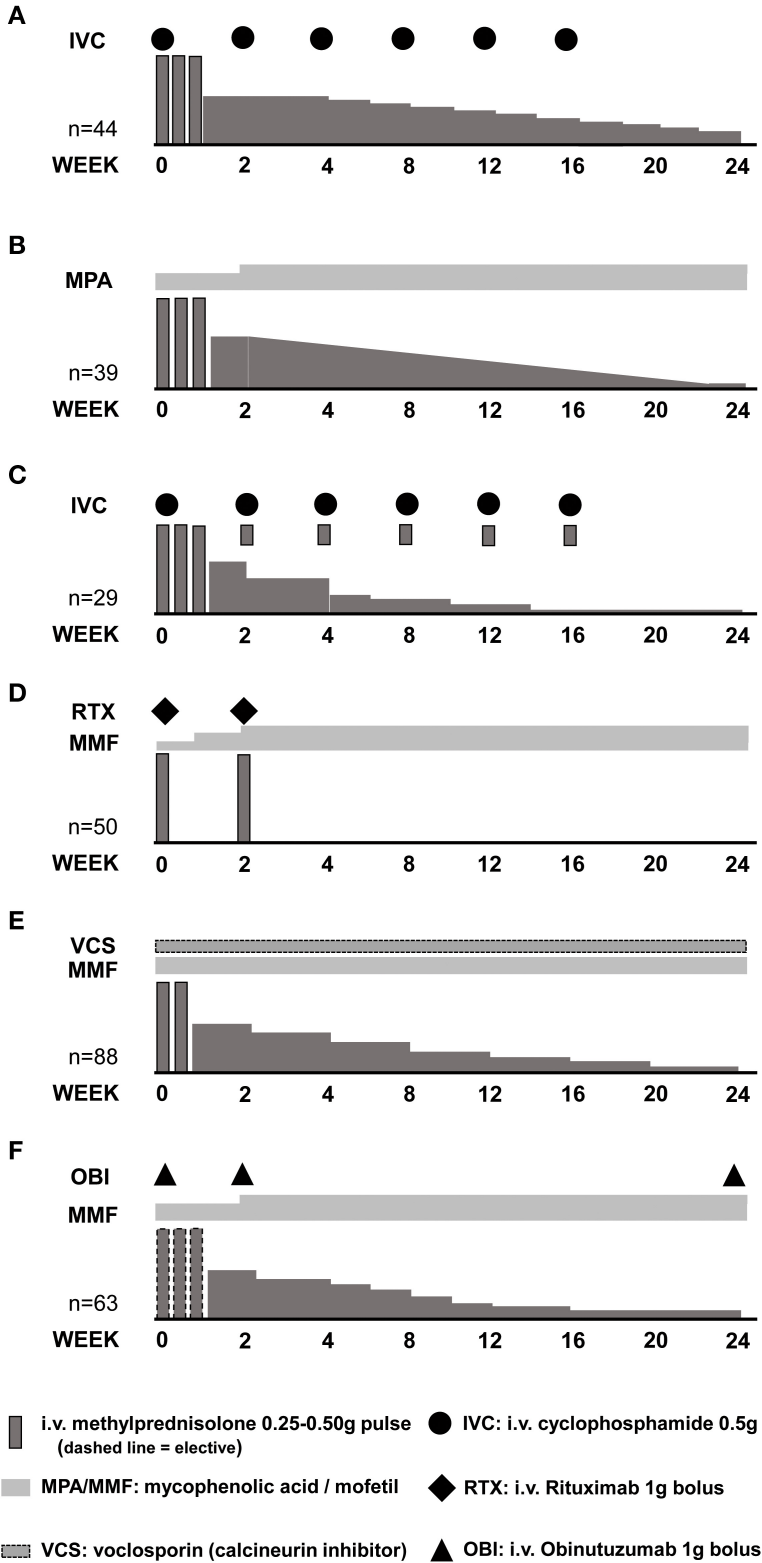
Induction of remission schemes in several studies. **(A)** Euro Lupus Nephritis Trial low-dose cyclophosphamide arm; **(B)** MYLUPUS reduced-dose glucocorticoid arm; **(C)** Lupus-Cruces protocol; **(D)** RITUXILUP protocol; **(E)** AURA-LV study voclosporin-treated arm; **(F)** NOBILITY study obinutuzumab-treated arm.

Administration of methylprednisolone pulses may allow the use of lower initial oral GC doses (lower peak GC dose) with a faster tapering schedule (lower exposure to high GC doses). Several clinical studies in lupus nephritis have included methylprednisolone pulses, followed by moderate ( ≤0.5 mg/kg/day) doses of oral GCs (Figure 3) (76–78). The Euro Lupus Nephritis Trial (ELNT) scheme (77) included three 750 mg methylprednisolone pulses, followed by 0.5 mg/kg/day prednisone slowly tapered to 10 mg/day by 6 months. This trial reported renal response rates (complete and partial) around 20 and 50% at 6- and 12-months, respectively, and long-term preservation of kidney function (77, 79).

The MYLUPUS trial (80) is the only randomized clinical trial that compared the efficacy of medium-dose oral GC therapy to high-dose GC. In this trial, all patients received three 0.5 g methylprednisolone pulses plus extended-release mycophenolate acid. Subjects were randomized to either high-dose oral GC scheme (starting dose 1 mg/kg/day) or to a reduced-dose oral GC scheme (starting dose ≈0.5 mg/kg/day). Complete and total response rates were similar at 6 months, 19 vs. 21% and 67 vs. 56%, respectively, in both groups.

In a trial evaluating a combination of calcineurin inhibitor, mycophenolate mofetil and GCs vs. intravenous cyclophosphamide, all patients received three 0.5 g/day methylprednisolone pulses followed by 0.6 mg/kg oral prednisone slowly tapered to 10 mg/day by week 16. Response rates of 84 and 63% at 6-months, were documented in the multi-targeted therapy and cyclophosphamide groups, respectively, and of 78% in both groups by 2 years of therapy (81–83).

More recent clinical trials have used lower doses of methylprednisolone pulses combined with a lower and faster oral GC tapering. In the AURA-LV (84) and AURORA (NCT03021499) trials evaluating combination therapy of voclosporin (a novel calcineurin inhibitor) with mycophenolate mofetil and oral GC, patients were treated with two 0.25–0.5 g methylprednisolone pulses, followed by a fixed 20–25 mg/d starting oral prednisone rapidly tapered to 5 mg by 12 weeks. Among clinical trials in LN, these two trials used the lowest peak oral GC doses and the faster tapering (Table 2). At 12 months, complete and total renal response rates of 49 vs. 24%, and 67 vs. 48%, respectively, were observed in the multi-targeted treatment and control groups in the AURA-LV trial (84).

**Table 2:** Estimated cumulative glucocorticoid doses in a 24-week period for a 60 kg patient in different induction to remission schemes.

**Regimen**	**Methylprednisolone total cumulative dose (g)**	**Oral prednisone total cumulative dose (g)**	**Oral prednisone average dose (mg/day)**	**Total GC dose (g)**
Modified NIH, 2001 (76)	9.00	2.84	16.9	11.8
ELNT, 2002 (77)	2.25	3.12	18.5	5.37
ALMS, 2009 (85)	–	4.27	25.4	4.27
MYLUPUS, 2011 (80)	1.50	2.14	12.7	3.64
RITUXILUP, 2013 (86)	1.00	–	–	1.00
LupusCRUCES, 2014 (48)	1.50-3.00	1.30-1.50	8.0-9.0	2.80–4.50
Chinese multitarget, 2015 (81)	1.5	3.25	16.2	4.75
4+2 Rituximab, 2015 (87)	2.70	2.52	15.0	5.22
AURA-LV, 2019 (84)	1.00	1.33	7.9	2.33
BLISS-LN, 2020 (88)	0.50–3.00^*^	3.12–4.27	18.5–25.4	3.12–4.27
NOBILITY, 2020 (89)	0.75–3.00^*^	1.79–1.93	10.6–11.5	1.79–1.93

* *Methylprednisolone pulses elective at discretion of the investigator*.

Uncontrolled single center experiences also suggest that treatment with methylprednisolone pulses allows a safe administration of lower starting oral GCs, and a faster tapering without compromising response and possibly reducing adverse effects. For example, the “Lupus Cruces” protocol for class III or IV LN includes the administration of three methylprednisolone pulses between 0.25–0.50 g, and an extra pulse of 0.1 g along with each cyclophosphamide bolus, following the ELNT scheme. The starting oral GC in this protocol was below 30 mg per day. In two reports, including 15 and 29 patients, response rates of 60 and 80%, and 86 and 87%, have been achieved at 6- and 12-months, respectively, with relapse rates below 15%. More importantly, the incidence of GC-related adverse effects was reduced to 7%, a significantly lower percentage when compared to that of historical or concurrent cohorts treated with higher doses of oral GC (48, 90).

Therefore, as evidenced in clinical trials and single-center experiences, the use of methylprednisolone pulses may allow reducing the starting oral GC doses and the duration of the exposure to high GC doses by allowing a faster tapering.

## The Use of “Reduced-Dose” Intravenous Methylprednisolone Pulses

There are no specific reports evaluating the dose of methylprednisolone in lupus nephritis. Although the use of methylprednisolone pulses has been associated with higher risk of infection in some cohort studies (32), these studies do not control for methylprednisolone dose. A small clinical trial including 21 patients with SLE (6 of them with nephritis) suggested that clinical outcomes are similar when using three daily 100 mg vs. 1 g methylprednisolone pulses. However, this trial did not control for other important variables such as concomitant treatment (91). While quality evidence is still low to support lower doses of methylprednisolone pulses, pharmacologic studies suggest that pulse doses above 0.5 g provide little additional anti-inflammatory benefit, and as mentioned earlier, may be associated with a higher incidence of adverse effects.

## Medium-Dose Glucocorticoid In Combination With New Immunosuppressant Drugs And Biologics

Combination therapy of mycophenolate mofetil, calcineurin inhibitors, and glucocorticoids may facilitate the use of lower GC doses. As previously mentioned, the AURA-LV trial used a forced reduced steroid taper along with mycophenolate ± voclosporin. The multi-targeted group showed 67% response rate by 12 months of treatment (84).

A recent trial evaluated the combination therapy of obinutuzumab, a novel B-cell depleting therapy, with mycophenolic acid analogs. All patients received a starting oral prednisone dose of 0.5 mg/kg with a fast taper to 7.5 mg by week 12, and optional methylprednisolone pulses. This trial has reported 52-week CR rates of 35%, maintained at 40 and 41% by 76 and 104 weeks, respectively (89).

Other immunosuppressants such as Janus kinase inhibitors, spleen tyrosine kinase inhibitors and biologics such as anifrolumab and ustekinumab, are being tested in patients with LN. Their addition might also facilitate the use of lower dose glucocorticoids in the future.

## Schemes Free of Oral Glucocorticoids

After initial reports describing the potential use of rituximab without increasing GC dose in renal (92) and non-renal lupus (93), the UK group from the Imperial College in London reported their first 50 patient experience with the RITUXILUP scheme (86). This regimen consists of 2 doses of rituximab 1 g administered with 0.5 g methylprednisolone followed by mycophenolate mofetil and no oral glucocorticoids. The initial report, which included class III, IV, and V LN patients, showed 6-month complete and total response rates of 32 and 62%, respectively. During follow-up, kidney function was preserved in most patients, with 22% of patients experiencing nephrotic relapses. Importantly, unlike the LUNAR trial (94) that failed to demonstrate a benefit of added rituximab to the standard of care therapy, depletion of B-cells to <5 B lymphocytes/mL was achieved in 93% of patients. The importance of B-cell depletion is supported by a sub-analysis from the LUNAR trial showing that complete response was more frequent in those subjects with B cell depletion (95). Therefore, although not yet demonstrated in a clinical trial, the RITUXILUP scheme supports the concept that the use of biologic drugs may facilitate the administration of GC free regimen in some patients with LN.

Targeting the activated complement system with complement inhibitors may also promote GC-reduced or GC-free regimens. Although complement inhibition in lupus nephritis has been used in a few case reports (96, 97), particularly in the context of concomitant thrombotic microangiopathy, the CLEAR (98) and ADVOCATE (99) studies in ANCA-associated vasculitis suggest this may be an approach worth investigating in lupus nephritis. In these studies, administration of avacopan (an oral complement C5aR inhibitor) along with cyclophosphamide or rituximab, allowed the administration of a GC-free regimen with higher remission rates at 52 weeks of follow-up in patients with ANCA-associated vasculitis (99).

## Concomitant Use of Antimalarials

The use of antimalarial in all patients with SLE and lupus nephritis is recommended in recent guidelines (38, 39). Although unexplored in controlled trials, combination schemes with antimalarial may add to the use of lower doses of GC by an enhanced effect for remission (100–102). Other demonstrated benefits from antimalarial, as the protective effect for damage accrual (103, 104), infections (33), and mortality (105), may add to the potential benefit of GC-reduced regimens.

## Glucocorticoids During The Maintenance Phase of Treatment

Maintenance therapy in lupus nephritis aims to consolidate the response obtained after the induction phase of therapy, and to prevent systemic and renal relapses. Current guidelines suggest tapering glucocorticoids to “the lowest possible dose” and to consider discontinuation after 12 months of complete remission (39).

Although there is no solid evidence in lupus nephritis, the CORTICOLUP trial (106) evaluated discontinuation of steroid in stable SLE patients (34–41% had history of LN). In this trial, patients receiving 5 mg of prednisone who have been stable for 1 year (the median quiescence duration was ≈5 years) were randomized to suspend or continue prednisone at the same dose. Disease flares were observed in 27% of patients who suspended prednisone vs. 7% in those who continued prednisone at 5 mg per day (RR 0.2, 0.01–0.7, *p* = 0.003). Only 3 patients had renal flares and the study was underpowered to evaluate the subgroup of patients with LN. Noteworthy, there were no differences in adverse events or damage accrual in both groups measured using the glucocorticoid toxicity index (107) and the SDI, respectively.

This study suggests that a low-dose of glucocorticoids at 5 mg per day may be safe and keeps patients free from disease flares. In other studies, the longer duration of the GC therapy before suspension has also been associated with less disease flares (108). A recent EULAR expert consensus suggested that at ≤ 5 mg/day, there is a low level of harm related to GC's main adverse effects (109), however, acknowledges that the actual risk of harm is patient-specific. Therefore, long-term glucocorticoid therapy must be balanced individually considering individual risk factors for flares (e.g., partial instead of complete response, persistently low C3), against individual risk factors for GC related adverse effects (e.g., age, cumulative GC dose, cardiovascular risk factors, presence of metabolic disease, etc.).

## Strategies To Minimize Corticosteroids During The Maintenance Phase

### Antimalarial Treatment

Antimalarials have been associated with lower incidence of disease flares in several observational cohort studies (110, 111). Moreover, reports of successful withdrawal of therapy in SLE patients have repeatedly found antimalarial treatment and duration of remission as the main factors associated with decreased odds of flares (112, 113). Also, as previously mentioned, antimalarials may reduce long-term damage from the disease activity (114).

## Biologics For Maintenance Therapy

Although evidence is still scarce, there is growing data suggesting that the use of certain biologics during the maintenance phase may aid in achieving sustained remission. For patients already on glucocorticoids, the RITUXIRESCUE regimen includes the administration of rituximab and methylprednisolone without increasing oral GC dose. This regimen showed a response rate of 78% in LN relapses, furthermore it allowed reduction or discontinuation of oral GC in more than 50% of patients during follow up (92).

An Italian strategy consisting of four 375 g/m^2^ rituximab doses reinforced by two additional doses at 1 and 2 months after (the 4+2 rituximab scheme), showed no flares during follow-up without the need for additional maintenance therapy beyond 5 mg of prednisone per day (87). Other small reports have highlighted the potential role of rituximab as a maintenance drug allowing glucocorticoid suspension (115). Therefore, although rituximab has not been tested for maintenance in a clinical trial, its use may aid in preventing flares during GC withdrawal.

In the BLISS-LN trial, the addition of belimumab to standard of care therapy (MMF or cyclophosphamide plus GC) showed a better response and a stable glomerular filtration rate beyond the induction phase, for up to 2 years of follow up (88). Furthermore, there have been small reports (116–118) suggesting that belimumab therapy may allow reduction or suspension of maintenance GCs, but this remains to be further studied.

Likewise, the NOBILITY trial has reported that the addition of obinutuzumab to standard of care therapy favored a sustained response, better glomerular filtration rate, and better serological profile at 76 weeks and onwards (89). This suggests that B cell targeted therapy may potentially facilitate GC withdrawal or at least a safe reduction to <5 mg/d of prednisone.

## Future Steps and A Word of Caution

Although recent advances in drug development in lupus nephritis promote the use of lower glucocorticoid doses, we must acknowledge that “one size does not fit all” patients. For example, patients with severe lupus nephritis presenting with a glomerular filtration rate below 30 mL/min/1.73 m^2^ have been excluded from most clinical trials, and there are no data to support the effectiveness of reduced glucocorticoid doses in this group of patients. Moreover, many of the published studies are single-center and observational reports subject to bias. Therefore, caution and case-by-case evaluation is recommended in selecting an appropriate glucocorticoid therapy.

Future studies in lupus nephritis will likely aim at using the lowest effective dose of glucocorticoids or glucocorticoid-free regimens. Studying the safety and efficacy of calcineurin inhibitors, biologic drugs or perhaps complement inhibitors in combination with standard of care therapy might lead successfully to this aim.

## Conclusions

The anti-inflammatory properties of GC have always been counterbalanced by their side effects. Adverse effects may be associated with peak doses, time under high doses, or cumulative doses. An objective for current and future management of lupus nephritis is to develop strategies that increase response to therapy with the least glucocorticoid exposure.

## Author Contributions

JM-V and IA designed the concept, planned, and performed this work.

## Conflict of Interest

The authors declare that the research was conducted in the absence of any commercial or financial relationships that could be construed as a potential conflict of interest.
